# Characterization of *Staphylococcus* Species Isolated from Bovine Quarter Milk Samples

**DOI:** 10.3390/ani9050200

**Published:** 2019-04-27

**Authors:** Regina Wald, Claudia Hess, Verena Urbantke, Thomas Wittek, Martina Baumgartner

**Affiliations:** 1 Department of Farm Animal and Public Health in Veterinary Medicine, University Clinic for Ruminants, University of Veterinary Medicine, 1210 Wien, Austria; verena.urbantke@vetmeduni.ac.at (V.U.); thomas.wittek@vetmeduni.ac.at (T.W.); martina.baumgartner@vetmeduni.ac.at (M.B.); 2 Department of Farm Animal and Public Health in Veterinary Medicine, University Clinic for Poultry and Fish Medicine, University of Veterinary Medicine, 1210 Wien, Austria; claudia.hess@vetmeduni.ac.at

**Keywords:** *Staphylococcus aureus*, coagulase-negative staphylococci (CNS), mastitis, colonizer, minimal inhibitory concentration (MIC)

## Abstract

**Simple Summary:**

Staphylococci are the most prevalent bacteria isolated from bovine mammary secretions. They not only originate from cases of intramammary infections, but also from teat canal, skin and other environmental sources. They are usually divided into coagulase-negative staphylococci (CNS) and *Staphylococcus (S.) aureus*. In contrast to the contagious nature of most *S. aureus* infections, the epidemiology of CNS is less clear. Results of our observational study suggest that both, CNS and *S. aureus*, can be associated with clinical and subclinical mastitis but may also appear as colonizers and remain undetected in cows without inflammatory signs. As a result, the consequences differ, especially with the increased emphasis on reducing antibiotic use as a means of limiting antimicrobial resistance (AMR). A positive *S. aureus* test result requires antibiotic treatment of infected cows after evaluation of the probability of bacteriological cure, and, where necessary, implementation of management strategies to limit new infections. In contrast, treatment of CNS in cows without increase in somatic cell count should be avoided. Thus, these findings emphasize the value of regular bacteriological examination of clinical and subclinical cases and a thorough evaluation of any staphylococcal presentation before treatment.

**Abstract:**

*Staphylococcus (S.) aureus* is considered as a major mastitis pathogen, with considerable epidemiological information on such infections while the epidemiology of coagulase-negative staphylococci (CNS) is more controversial. The aim of this study was to use matrix-assisted laser desorption ionization time-of-flight mass spectrometry (MALDI-TOF MS) technology for identification of staphylococci isolated from bovine milk at species level and to characterize them in reference to presentation, somatic cell count (SCC), bacterial shedding (cfu) and antimicrobial resistance patterns. A total of 200 staphylococcal isolates (*S. aureus n* = 100; CNS *n* = 100) originating from aseptically collected quarter milk samples from different quarters of dairy cows were included in the study. They originated from cases of clinical (CM) and subclinical mastitis (SCM) or were isolated from milk with SCC ≤ 100,000 cells/mL in pure culture. We found staphylococci predominantly in cases of SCM (*n* = 120). In low-SCC cows, 12 *S. aureus* and 32 CNS isolates were detected. Eighteen percent of each were associated with CM. Eleven CNS species were identified, *S. chromogenes* (*n* = 26) and *S. xylosus* (*n* = 40) predominated. CNS, particularly those in low-SCC cows, showed higher MIC90 (minimal inhibitory concentration) values for penicillin, ampicillin, cefoperazone, pirlimycin and marbofloxacin. Based on the present results, a careful interpretation of laboratory results is recommended to avoid antimicrobial therapy of staphylococci without clinical relevance and to ensure prudent use of antimicrobials.

## 1. Introduction

Mastitis is the main reason for antimicrobial treatment in dairy cattle. Pathogen specific treatments of mastitis have been shown to decrease antimicrobial use without affecting clinical and bacteriological cure outcomes [1,2]. A major factor in obtaining a bacteriological cure of intramammary infections (IMI) is an accurate diagnosis [3]. IMI are detected frequently through milk culturing. Microbial culturing of quarter milk samples enables the monitoring bovine udder health as well as identification of etiological agents and their antimicrobial susceptibility, which ensure targeted antimicrobial therapy [4]. Strict aseptic sampling techniques and proper storage of milk samples are essential to avoid false positive results. Regular monitoring of mastitis pathogens within a herd facilitates treatment decisions in case of clinical mastitis [5,6]. Due to the time delay between sampling and culture results, treatment of mastitis is usually based on bacteriological examination of a single sample. Factors involved in diagnosing an IMI are the number of colonies, isolation in pure or mixed culture, and inflammatory signs [4,6].

Staphylococci are the most commonly isolated bacteria from milk of dairy cows [7,8,9]. In routine mastitis diagnosis, staphylococci are usually divided either into coagulase-negative staphylococci (CNS)/non-*aureus* staphylococci (NAS) and coagulase-positive staphylococci/*Staphylococcus (S.) aureus* [7]. CNS are a heterogeneous group with more than 15 species having been isolated in association with bovine mastitis [9,10,11]. The accurate identification of CNS at species level cannot be provided reliably by classical bacteriology using phenotypic and biochemical criteria alone [12]. While *S. aureus* is considered as a major pathogen whose primary mode of transmission is cow-to-cow, the epidemiology of CNS mastitis is still unclear [7,12,13]. CNS are considered as opportunistic pathogens causing mastitis. Similar to *S. aureus*, various CNS species have been isolated from extramammary sites such as bovine skin and teats [7,14,15]. Some authors suggest that some CNS strains as a native part of a microflora may play a positive role in the maintenance of udder health [16]. These facts have to be considered in regard to the promotion of a prudent use of antimicrobials, as in such cases these bacteria can be recovered from milk samples of dairy cows without any apparent increase in somatic cell count (SCC) [17,18,19].

CNS have generally been regarded as most likely to cause only a slight increase of SCC and a mild self-limiting clinical inflammatory response, particularly in heifers. However, some CNS can mimic *S. aureus*, causing both clinical and subclinical mastitis, which often remains persistent [8,14,18,20]. Recent studies propose that infections with CNS may cause more serious harm than previously thought [21]. To date, the classification of staphylococci into *S. aureus* and CNS has been considered as sufficient for managing bovine mastitis in the field [7]. However, recent research has questioned this [7,9,17,22].

Thus, the aim of this study was to differentiate *Staphylococcus* isolates from aseptically collected quarter milk samples by using matrix-assisted laser desorption ionization time-of-flight mass spectrometry (MALDI-TOF MS), a technology that has been proven as a rapid, accurate and high throughput method for the differentiation of CNS [23,24]. As well as analysis of different species with regard to presentation, SCC, bacterial shedding and antibiotic resistance profile, the study was accompanied by a questionnaire on veterinary practices associated with *Staphylococcus* spp. positive culture results. The hypothesis was that some species are more prone to interact as non-pathogenic colonizers than others and that positive bacteriological culture results lead veterinarians to initiate an antimicrobial treatment that may be unnecessary.

## 2. Materials and Methods

### 2.1. Study Design

The study period was carried out for seven months between March and September 2017. Staphylococcal isolates originated from aseptically collected quarter milk samples sent for bacteriological culturing to the laboratory of the University Clinic for Ruminants in Vienna (Austria). This includes samples taken during farm visits during our routine activities, standard culturing of in-house patients in the clinic as well as samples sent from farms for bacterial culture delivered via medical shipping companies (convenience sampling). The goal was to collect 100 isolates each (*S. aureus* and CNS). We terminated the collection when the number of isolates was achieved. In detail, from March to September 2017, quarter milk samples of 3142 cows were analyzed, and 666 quarters have been positive for *S. aureus* and 979 quarters for CNS. To avoid inclusion of staphylococcal contaminants, strict criteria were applied comprising growth in pure culture and a complete medical history form, which provided information on the sampled cow (affected quarter, increased SCC, abnormal milk, swelling of quarter, fever, teat laceration, check after treatment). To gain isolates from presumptive healthy glands, control examinations including a routine post-calving check and control check prior to either drying-off or before auction were included.

### 2.2. Laboratory Examination and Bacterial Isolates

Ten microliters of each milk sample was plated onto Columbia agar supplemented with 5% sheep blood (Oxoid Ltd., Basingstoke, UK) and incubated at 37 °C. After 24 h, isolates were selected based on their growth characteristics as phenotypically identical colony types in pure culture on the primary plate. Isolates were identified as *S. aureus* or CNS based on phenotypical and biochemical criteria as suggested by the National Mastitis Council (NMC) [20]. Appearance on blood agar and hemolytic patterns have been assessed. All gram-positive, catalase-positive, and coagulase-positive cocci were considered *S. aureus*. Isolates originated from different quarters from 163 cows from 142 herds located in the following Austrian federal states: Burgenland (*n* = 3), Lower Austria (*n* = 106), Salzburg (*n* = 70), Styria (*n* = 19), Tyrol (*n* = 2). All bacteria were stored at −80 °C in a 15% glycerol solution for confirmation and identification to species level by applying MALDI-TOF MS (Bruker Daltonics, Bremen, Germany). For this purpose, each isolate was cultured on sheep blood agar at 37 °C for 24 h. Colonies were spotted in duplicate by direct transfer method on to MALDI-TOF MS target plate, allowed to dry, and overlaid with 1 µL of matrix solution (α-cyano-4-hydrosycinnamic acid in 50% acetonitrile and 2.5% trifluoroacetic acid) and allowed to dry. Generated spectra were assigned a log(score) based on similarity with the spectra from the MBT Biotyper database (Bruker Daltonics): log(score) values ≥ 2.000 correspond to genus and species identification with high confidence, log(score) values between 1.700 and 1.999 to genus identification only, and log(score) values lower 1.700 are rated as no reliable identification. Isolates which could not be identified by using the direct transfer method were investigated by using the ethanol/formic acid extraction method according to the manufacturer’s protocol. In brief, one single colony was mixed with 300 µL HPLC-grade water. Afterwards, 900 µL of pure ethanol was added and mixed. The solution was spun and the supernatant was removed. This step was repeated two times. Then the pellet was allowed to dry for 15 min, and mixed with 50 µL of 70% formic acid and 50 µL of acetonitrile, spun and 1 µL of the supernatant was spotted on the MALDI-TOF MS target plate. The material was allowed to dry and overlaid with 1 µL of matrix solution. The run was identical as mentioned before. Furthermore, a PCR assay specific for *S. aureus* by targeting the *nuc* gene with primers nucA1 (5′-GCGATTGATGGTGATACGGTT-3′) and nucA2 (5′-AGCCAAGCCTTGACGAACTAAAGC-3′) according to Brakstad et al. [25] was used. In short, cycling conditions were as follows: 94 °C for 2 min, followed by 30 cycles of 94 °C for 30 seconds, annealing at 55 °C for 30 seconds, elongation at 72 °C for 90 seconds, and a final elongation at 72 °C for 3.5 min. Reaction products were visualized on 1.5% agarose gel electrophoresis (1× TBE buffer, 120 V, 30 min).

### 2.3. Determination of Colony Forming Units and Somatic Cell Count

The bacterial shedding (colony forming units—cfu) was estimated by using the plate drop method [26]. In brief, serial dilution of 25 µL milk and 225 µL sterile saline solution (0.9% NaCl, B. Braun, Melsungen, Germany) was done in which, the dilution of 1× suspension was added to 9× of diluent. Six dilution steps were made. Starting with highest dilution, three drops of 25 µL of each dilution step were placed on Columbia blood agar and incubated at 37 °C for 24 h. The colonies of the lowest dilution were counted and the total count (cfu/mL) was scaled up. Additionally, SCC in single quarter milk samples was measured with DeLaval cell counter DCC (Tumba, Sweden).

### 2.4. Antimicrobial Resistance Testing

Antimicrobial resistance (AMR) was tested using a commercially available minimal inhibitory concentration (MIC) microtiter plate assay. In MICRONAUT-S Mastitis 3 (Merlin Diagnostika, Bornheim, Germany), a panel of 11 antimicrobials at the following concentrations were included: amoxicillin/clavulanic acid (4/2–32/16 µg/mL), ampicillin (4–16 µg/mL), cefazolin (4–32 µg/mL), cefoperazone (2–16 µg/mL), cefquinome (1–8 µg/mL), erythromycin (0.25–4 µg/mL), kanamycin/cephalexin (4/0.4–32/3.2 µg/mL), marbofloxacin (0.25–2 µg/mL), oxacillin (1–4 µg/mL), penicillin G (0.125–8 µg/mL), and pirlimycin (1–4 µg/mL). Isolates were incubated on Columbia agar containing 5% sheep blood for 24 h at 37 °C. The overnight cultures were suspended in sterile saline solution (0.9% NaCl, B. Braun) to achieve a turbidity of McFarland standard 0.5, and then diluted 1:110 according to manufacturer’s instructions before use. Isolates were characterized as susceptible, intermediate or resistant based on breakpoints for staphylococci isolated from animals provided by the Clinical Laboratory Standard Institute (CLSI, Wayne, PA, USA) or published in reference works [27,28,29]. MIC50 and MIC90 were defined as the concentration of the antimicrobial agents able to inhibit the growth of 50% and 90% of the isolates in the test population, respectively [30]. Additionally, β-lactamase production testing of isolates was performed using nitrocefin-impregnated discs (Mast Diagnostica, Reinfeld, Germany) in accordance with the manufacturer’s instructions.

### 2.5. Categorization and Definitions

**Figure 1:**
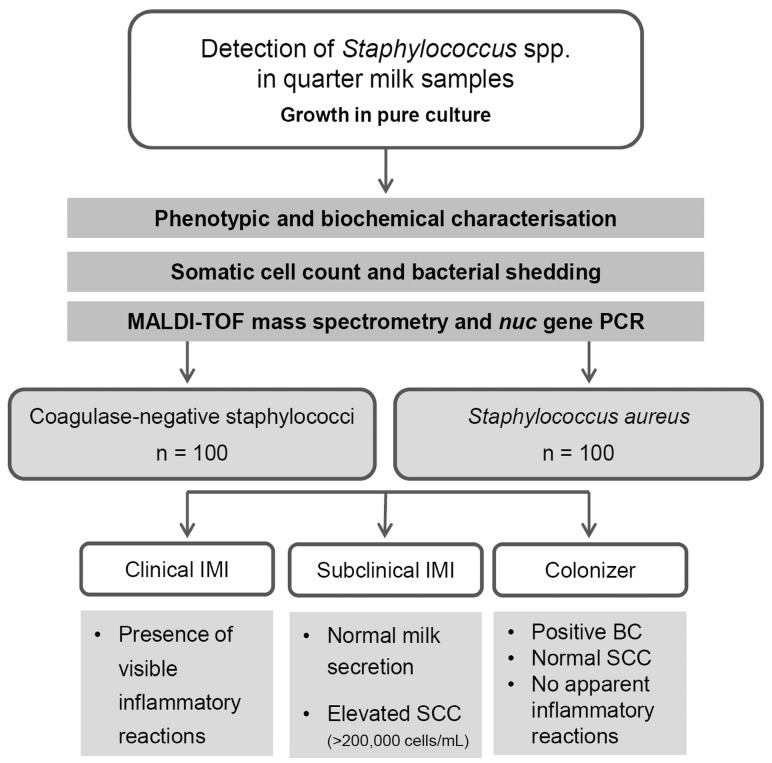
Study group formation of the staphylococcal isolates collected from March to September 2017 in routine diagnosis. (MALDI-TOF = matrix-assisted laser desorption ionization time of flight; PCR = polymerase chain reaction; IMI = intramammary infection; SCC = somatic cell count; BC = bacteriological culturing).

Isolates were assigned to three different categories (Figure 1): (a) subclinical mastitis (SCM) was defined by macroscopically normal milk accompanied by increased values of SCC (>200,000 cells/mL); (b) clinical mastitis (CM) was defined by the presence of visible inflammatory reactions like alterations of the milk sample (color, consistency) or anamnestic report of systemic (fever) or local inflammatory reactions (e.g., quarter swelling, abnormal milk secretions); (c) the term colonizers is used to define isolates originating from low-SCC milk samples. They were obtained from low-SCC cows based on a single point-in-time sampling where no apparent inflammatory changes of the mammary gland were reported, no changes in macroscopic milk appearance were observed and a physiological SCC (≤100,000 cells/mL) was measured [6,31].

### 2.6. Questionnaire

Data about mastitis treatment regimes with regard to the interpretation of microbiology results and antimicrobial use by veterinary practitioners were collected. The hyperlink to an anonymously internet-based survey focusing on CNS and *S. aureus* was sent to practitioners who commission bacteriological examinations of quarter milk samples. The survey included five multiple choice questions and a further comments section to gather information about clinic-specific management practices and specifications of methods. The questionnaire (Table S1) was designed according to the following principles: Treatment of cows I. with clinical mastitis; II. CNS or *S. aureus* in cows with subclinical mastitis and check-ups except control prior to drying-off; III. CNS or *S. aureus* in controls prior to drying-off. Specification on antimicrobial agents were provided voluntarily in a free-text field.

### 2.7. Statistical Analysis

All statistical analyses were performed using the software SPSS Statistics 23.0 (IBM, Armonk, NY, USA) and Microsoft Excel 2013 (Microsoft Cooperation, Redmond, WA, USA). For all tests, statistical significance was defined at *p* ≤ 0.05.

SCC was summarized into four classes (≤100,000; 200,000–500,000; 500,000–1 million; >1 million cells/mL). Results of bacterial shedding were divided into four groups (≤10^2^; 10^3^–10^4^; 10^5^–10^6^; ≥10^7^ cfu/mL). Individual cow SCC and cfu were transformed into log counts.

Data were tested for normal distribution by the Kolmogorov-Smirnov test. One-way analysis of variance (ANOVA) including Bonferroni post-hoc test was applied to analyze the differences between the *Staphylococcus* species in SCC and bacterial shedding and Pearson coefficient was used to determine the correlation with SCC and cfu. Differences in presentation were tested using a chi-squared test.

## 3. Results

### 3.1. Phenotype, AMR and IMI Characteristics of S. aureus

*S. aureus* isolates (*n* = 100) originated from different quarters of 82 cows (67 cows = 1 quarter, 12 cows = 2 quarters, 3 cows = 3 quarters) kept on 66 different dairy farms. All *S. aureus* isolates were confirmed by nuc gene PCR and MALDI-TOF MS. A double-zone hemolysis (both complete and incomplete hemolysis) was induced by 61 field isolates and 37 isolates were classified as β-hemolytic. No hemolysis was found in two *S. aureus* isolates.

The majority of our *S. aureus* isolates (*n* = 70) were derived from cases of subclinical mastitis; 18 isolates were associated with clinical mastitis and twelve isolates were characterized as colonizers. A clear difference in the median cfu group of quarter milk samples could be seen between subclinical mastitis and CNS in low-SCC cows both containing 10^3^–10^4^ cfu/mL compared to clinical mastitis where 10^5^–10^6^ cfu/mL were present (Table 1). No correlation between cfu values and SCC values could be found by applying Pearson’s correlation (r = 0.029; *p* = 0.781).

AMR testing demonstrated that none of the *S. aureus* field isolates were resistant against kanamycin/cephalexin. They were mostly susceptible to cefoperazone (90%), penicillin (90%) and oxacillin (98%). Six of the tested isolates produced β-lactamase and were simultaneously resistant to penicillin. More than half of the isolates (59%) demonstrated in vitro susceptibility to pirlimycin. Intermediate or fully resistant results to erythromycin were found in 37% and 4% of isolates, respectively. In general the isolates had low MIC90 values for ampicillin, amoxicillin/clavulanic acid, cefazolin (≤4 µg/mL each) and cefquinome (≤1 µg/mL). Marbofloxacin proved less active against *S. aureus* with a MIC90 value of 0.5 µg/mL (Table 2). Due to the low prevalence (12%) of *S. aureus* in low-SCC cows, a MIC90 was not calculated. All these isolates displayed hemolysis on blood agar and were inhibited in growth by the lowest tested concentration of cefazolin, cefquinome, oxacillin and amoxicillin/clavulanic acid. None of the twelve isolates were positive for β-lactamase production.

### 3.2. Phenotype, AMR and IMI Characteristics of CNS

According to phenotypical and biochemical criteria, 100 isolates were identified as CNS originating from different quarter samples of 81 cows (66 cows = 1 quarter, 11 cows = 2 quarters, 4 cows = 3 quarters) from 76 dairy farms. Broad variations in colony morphology and color in this group of bacteria were observed. At species level, CNS isolates were identified by MALDI-TOF MS as *S. xylosus* (*n* = 40), *S. chromogenes* (*n* = 26), *S. haemolyticus* (*n* = 7), *S. sciuri* (*n* = 5), *S. simulans* (*n* = 3), *S. succinus* (*n* = 3), *S. saprophyticus* (*n* = 3), *S. epidermidis*, *S. equorum*, *S. hyicus* and *S. intermedius* (one isolate each). Nine CNS isolates could not be classified to the species level (log(score) value 1.700–1.999). For most of the cows (*n* = 12/15) with isolates obtained from multiple quarters, the same CNS species per cow was confirmed. The majority of CNS did not induce hemolysis on blood agar but all *S. haemolyticus* and *S. intermedius* isolates and further eight isolates (5 *S. xylosus*, 2 *S. simulans*, 1 *Staphylococcus* spp.) had β-hemolysis. All convex mucoid growing isolates (*n* = 6) were identified as *S. chromogenes*. *S. sciuri* (*n* = 5) displayed flat greyish-translucent colonies with a centered white brightening.

The majority of our isolates (*n* = 68) could be linked to IMI, whereas 32% of CNS belonged to the category of colonizers. As with the results of *S. aureus*, the majority of isolates (*n* = 50) could be attributed as the cause of subclinical mastitis. Eighteen isolates were associated with clinical mastitis. No difference could be found in regard to the median cfu group in regard to the presentation (Table 1). As already stated for *S. aureus*, no Pearson’s correlation (r = −0.138; *p* = 0.172) could be found between cfu and SCC.

The CNS causing IMI were mainly *S. xylosus* (24/40) and *S. chromogenes* (18/26) and were predominantly associated with a subclinical presentation (Table 3). ANOVA revealed no significant differences in cfu values or SCC values between the different CNS species.

AMR testing (Table 4) showed that most of the CNS isolates were susceptible to kanamycin/cephalexin (98%). Resistance to penicillin, erythromycin and cefoperazone was found in 17%, 7% and 4% of isolates, respectively. In particular a high number of isolates showed intermediate resistance against erythromycin and cefoperazone (35% and 18%, respectively). CNS isolates were similar to *S. aureus* isolates, having low MIC90 values for ampicillin, amoxicillin/clavulanic acid, cefazolin (≤4 µg/mL each) and cefquinome (≤1 µg/mL). Ten field isolates were positive for β-lactamase production, but applying CLSI breakpoints, six of them were classified as susceptible to penicillin. Isolates associated with mastitis had lower MIC90 values compared to CNS in low-SCC cows for penicillin (0.25 vs. 4 µg/mL), ampicillin (<4 vs. 8 µg/mL) and pirlimycin (2 vs. >4 µg/mL).

### 3.3. Comparative Evaluation of S. aureus and CNS

Between *S. aureus* and CNS, chi-squared test revealed significant differences (*p* = 0.002) in presentation. In our data set, we found 32 CNS and 12 *S. aureus* isolates to act as colonizers (Table 1). Regarding SCC and cfu values, there was a significant difference found between *S. aureus* and CNS (ANOVA SCC *p* = 0.027 and cfu *p* < 0.001). Differences in cfu were found comparing *S. aureus* and *S. chromogenes* (Bonferroni correction *p* = 0.004). Mean bacterial shedding in samples with *S. chromogenes* was estimated with 5 × 10^4^ cfu/mL, in samples with *S. aureus* 6 × 10^7^ cfu/mL. For SCC, differences between *S. aureus* and *S. xylosus* were found (Bonferroni correction *p* = 0.016). Mean SCC in milk samples with *S. xylosus* was 515,000 cells/mL and in samples with *S. aureus* 1.1 million cells/mL.

Differences in in vitro susceptibility were found (Table 2 and Table 4). Compared to *S. aureus* isolates, MIC90 of CNS was higher for penicillin, cefoperazone and marbofloxacin. Except for *S. chromogenes*, CNS were generally more susceptible to erythromycin than *S. aureus*. For *S. aureus* and *S. chromogenes* isolates, lower MIC90 values for cefoperazone and oxacillin than the remaining CNS were observed. Despite similar susceptibility rates for kanamycin/cephalexin, MIC90 of *S. aureus* isolates was higher than that of CNS.

### 3.4. Survey

Questionnaires from 136 Austrian practitioners were completed which yielded more than 1100 evaluable responses. Data analysis showed that 92.7% (*n* = 127) of queried veterinarians treated cows with clinical mastitis immediately with antimicrobials (30.7% intramammary, 21.9% parenteral, 60.6% both) without any direct knowledge of the involved pathogens. Substances used (65 statements) are in descending order of nomination penicillins (*n* = 38: penicillin, amoxicillin, cloxacillin), cephalosporins (*n* = 29: cefquinome, cefoperazone, cephalexin), fluorchinolones (*n* = 15: enrofloxacin, marbofloxacin), macrolides (*n* = 7: tylosin), aminoglycosides (*n* = 4: kanamycin) and combination antimicrobials (*n* = 13: kanamycin/cephalexin, lincomycin/neomycin, amoxicillin/clavulanic acid, penicillin/gentamycin). Non-steroidal anti-inflammatory drugs (*n* = 10) or homeopathic products (*n* = 1) were also administered. For selecting the appropriate treatment, decisions were based on susceptibility testing results in the herd and clinical symptoms (*n* = 9). In case of subclinical mastitis and control examinations (except controls prior to drying-off) with CNS positive culturing, penicillins (*n* = 19), cephalosporins (*n* = 10), macrolides (*n* = 3), and combination antimicrobials (*n* = 6: kanamycin/cephalexin, lincomycin/neomycin, amoxicillin/clavulanic acid, tetracycline/neomycin) were used according to susceptibility testing (38 statements). About 42.6% of the surveyed veterinarians re-checked SCC again and treated only in case of elevation. Further results are presented in Table 5. At drying-off, the following antimicrobials (30 statements) were commonly used: β-lactams (*n* = 22: cloxacillin, cefquinome) and combination antimicrobials (*n* = 5) according to susceptibility testing. Most of the practitioners (86.0%) decided on an antimicrobial dry cow therapy when culturing reported CNS.

For *S. aureus*, a differentiated approach was emphasized (*n* = 14): Treatment decisions were based on mastitis history, chronicity, number of infected quarters, SCC, period of lactation, parity and herd-specific factors. Favored substances (31 statements) in case of subclinical mastitis and control examinations (except controls prior to drying-off) were β-lactams (*n* = 21: cloxacillin, penicillin, cefquinome), macrolides (*n* = 7) and lincosamides (*n* = 4) according to susceptibility testing. For cows at drying-off with *S. aureus* positive culture result, penicillins (*n* = 36: penicillin, cloxacillin), macrolides (*n* = 11) and cephalosporins (*n* = 6) were widely used according to susceptibility testing (39 statements). Vaccination against *S. aureus* mastitis was mentioned (*n* = 3) as well as culling (*n* = 6) or management practices like milking order (*n* = 2). The responding veterinarians stated that they treated (Table 5) only in case of increased SCC (26.5%) or they do not treat *S. aureus* mastitis during lactation (39.7%). Most veterinarians (81.5%) started an antimicrobial dry cow therapy if the sample tested positive for *S. aureus* irrespective of clinical signs or SCC.

## 4. Discussion

Bovine mastitis is the most important disease in dairy cattle production worldwide and is often classified as either clinical mastitis or subclinical mastitis [31,32]. Both presentations cause economic loss, mainly due to reduced milk production, disposal of milk, and treatment costs [33,34]. *S. aureus*, as well as CNS, are associated with IMI but can also be detected in mammary secretions of cows without increased SCC [17,19]. Furthermore, they can also be found on extramammary sites. Thus, it is important to be aware that organisms isolated from milk samples can act as pathogens, commensals or contaminants. This fact has to be taken into account in regard to the necessity of antibiotic treatments [4,5,6,19,35,36]. The species identification of CNS has often been neglected even though a greater understanding of these bacteria would possibly allow better management and control recommendations [12,18,37]. Therefore, we wanted to determine the species differences between the various staphylococci isolated from quarter milk samples with regard to their presentation and antibiotic resistance profile and to assess the therapy concepts of veterinarians regarding *Staphylococcus* spp. positive culture results.

The present investigation confirmed other reports that the majority of *S. aureus* isolates were found associated with subclinical mastitis but cases of *S. aureus* positive quarter milk samples with SCC values below 200,000 cells/mL were also found, again confirming other studies [14,18,19,38]. The latter could be categorized as colonizers or opportunistic pathogens. Intriguingly the present data revealed that the mean cfu groups of *S. aureus* in case of subclinical mastitis and in low-SCC cows were similar. Since our dataset results from a convenience sample, conclusions about prevalence outside the sampled population are limited. With regard to *S. aureus* control and management, it is important to consider that *S. aureus* colonization of the mammary gland may remain undetected for prolonged periods of time, especially if evaluation of udder health is mainly based on SCC data and/or in consideration of intermittent shedding, the determination of cfu values. In summary, any cow positive for *S. aureus* can be a source for transmission within a herd [39].

Antimicrobial therapy is part of *S. aureus* control programs [18,39]. Treatment decisions should be based on various cow factors and the knowledge of the antimicrobial sensitivity and manifestation [31,39]. McDougall et al. [40] observed a lower distribution of MIC for penicillin and cloxacillin from clinical cases in comparison to subclinical cases. Because of the relatively small test population in this study, no interpretation of MIC data for clinical and subclinical isolates was performed, but the majority of our *S. aureus* isolates showed low MIC values for all tested β-lactams. Pirlimycin and erythromycin were less active against several isolates, the aforementioned fact has also been reported recently in Austria and these findings should be kept in mind as the most commonly used antimicrobials for therapy are penicillin, erythromycin, tylosin and pirlimycin [2,39,41]. The questionnaire results revealed that Austrian veterinarians give regular consideration to susceptibility testing and follow a tailored approach when culturing detects *S. aureus*. At drying-off with an *S. aureus* positive culture, the participating veterinarians most commonly administer antimicrobials. Scherpenzeel et al. [42] found *S. aureus* more prevalent in low-SCC cows that did not receive dry cow antibiotics at drying-off and stated that quarters with a positive culture result for major pathogens at drying-off had a higher risk for an increased SCC two weeks after calving. Referring to this, antimicrobial dry-cow therapy in the case of *S. aureus* isolation can be justified, independent of SCC findings.

In contrast to the contagious nature of most *S. aureus* and constructive programs to control *S. aureus* IMI, the epidemiology of CNS mastitis and the impact of specific species is more controversial. When CNS are detected in quarter milk samples, identification of species may be relevant for mastitis control programs, management procedures and decisions on therapy strategies [12,14,43]. By applying MALDI-TOF MS, eleven different CNS species isolated from bovine milk samples were identified. Among these are the five major CNS species: *S. chromogenes*, *S. epidermidis*, *S. haemolyticus*, *S. simulans* and *S. xylosus* [44]. *S. chromogenes* and *S. xylosus* were predominantly found in the present investigation and this is in agreement with reports from Switzerland with small-scaled farms with an average herd size below 30 cows similar to Austria [45]. In general, the majority of our CNS bacteria could be seen in association with subclinical mastitis. Interestingly, approximately one third of our CNS isolates could be categorized into the group of colonizers but no CNS species was clearly associated with any one of the three categories (CM, SCM, colonizer). This finding is in accordance with the data from Persson Waller et al. [11], who found *S. chromogenes* and *S. xylosus* were not significantly associated with either clinical or subclinical IMI. In contrast to this, Taponen and Pyörälä [7] described that CNS species (except for *S. chromogenes*) isolated from teat skin, apex and canal mainly differ from the CNS species isolated from milk while De Visscher et al. [46] found that *S. chromogenes* and *S. xylosus* favored the mammary gland rather than the environment. The relative importance of different CNS strains is still open to debate, although this study adds to our information about them.

*S. aureus* and CNS strain differences with respect to transmissibility and pathogenicity have been described [8,13,47]. In the present study significant differences in SCC could be found between *S. aureus* and *S. xylosus*. Among *Staphylococcus* species, differences in in vitro activity have been observed [11,13,40]. Penicillin resistance of *S. aureus* has been described as generally lower than that of CNS [7]. The pan-European antimicrobial susceptibility monitoring reported that *S. aureus* had higher MIC90 values for amoxicillin/clavulanic acid and kanamycin/cephalexin than CNS, while CNS had lower MIC90 values for cefquinome and oxacillin [48]. In contrast to this, the German Resistance Monitoring stated similar MIC90 values for amoxicillin/clavulanic acid, cefquinome, cefoperazone and marbofloxacin [49]. Monitoring the antimicrobial resistance of *Staphylococcus* spp. in animals is not only important for treatment decisions in field, but from a public health perspective, because bacteria in the dairy environment can be a possible reservoir of resistance genes for both animals and humans [50]. Interestingly, CNS in our study, in particular those in low-SCC cows, have been found to be more resistant to some antimicrobials than *S. aureus*. This finding is in agreement with Taponen and Pyörälä [7] and Morin [18] who described that resistance to various antimicrobial agents is more common in CNS than in *S. aureus* and resistant CNS are more likely to be found on teats and in the environment. Systemic administration of penicillins, third-generation cephalosporins and macrolides are especially associated with higher resistance in CNS [51]. Typically, AMR increases first in the commensal flora and is transferred to pathogens afterwards [52]. For these reasons we and others consider that CNS in low-SCC cows should not be treated with antimicrobials because they may reflect teat canal colonization and could contribute to the spread of AMR [18,20,52,53]. The survey revealed that particularly prior to drying-off, SCC is rarely checked again but antimicrobial dry cow therapy is administered. It should be noted that in a Dutch study from drying-off to 14 days post-calving there were no significant differences in CNS prevalence found in low-SCC cows (<150,000 cells/mL for primiparous and <250,000 cells/mL for multiparous cows) with dry-cow antibiotics and those which did not receive antimicrobials [42]. With single point-in-time data, it can be difficult to determine whether the organism is truly colonizing the udder or streak canal, or was a contaminant; it may be argued that detection in subsequent sampling events would be more convincing for the definition of colonization. The current recommendation for considering a single quarter sample positive for an IMI is based on measuring the inflammatory response, detection of an organism in culture having regard to of colonies isolated and growth either in pure or mixed culture. Whatever the case, the attending veterinarian has to decide on the basis of a single culture result and detection of bacteria without inflammatory signs must be assessed critically regarding antibiotic use. Individual cow culture results become much more meaningful when combined with SCC results. For this purpose, SCC data collected monthly by dairies or California Mastitis Test scores should ideally be taken into consideration in the diagnosis of IMI in the field [6,18,35,39,54].

The present investigation provides data that bacteria belonging to the CNS group should be considered as agents that cause mastitis similar to *S. aureus*. Even though the results are in accordance with previous reports, the sample size and high variation between cows and herds has contributed to the lack of statistical significance between species. Supré et al. [43] found *S. chromogenes*, *S. simulans* and *S. xylosus* inducing an increase in the SCC that is comparable with that of *S. aureus*. Interestingly two of these, *S. chromogenes* and *S. xylosus*, covered two thirds of our CNS isolates. Our approach was limited as it did not include identification of risk factors such as cow, herd or days in milk. Similarly while the numbers of herds is high, the numbers of replicates within herd are low, which has implications on the reported outcomes regarding differences among *Staphylococcus* species. Irrespective of the species-specific impact of CNS, since phenotypic methods to differentiate among CNS yield unreliable results and mastitis control programs are not based on species level identification, “CNS” remain a frequent culture result in routine diagnosis and need to be considered in a clinical context [37,44].

## 5. Conclusions

*S. aureus*, as well as CNS, could be isolated from subclinical and clinical mastitis cases as well as from milk with SCC ≤100,000 cells/mL. In our sample pool, both were mainly detected in context with subclinical mastitis and CNS were more often found in low-SCC cows than *S. aureus*. AMR testing of some isolates demonstrated high MIC values for pirlimycin, erythromycin and marbofloxacin. With regard to prudent use of antimicrobials, it is necessary to avoid antimicrobial treatment of CNS without clinical relevance. These findings corroborate the importance for regular bacteriological examinations and thorough evaluation of staphylococcal isolations before treatment.

In summary, without this added depth of investigation of CNS species in the field, we recommend adhering to an aseptic sampling technique when samples are collected for culture and assessing culture results as here namely—clinical, subclinical and without clinical relevance.

## Figures and Tables

**Table 1:** *Staphylococcus aureus* and coagulase-negative staphylococci assigned to three different presentations including range and mean somatic cell count and colony forming units in quarter milk samples.

Isolates	Manifestation	*n*	Median cfu Group ^1^	Median SCC Class ^2^	Mean SCC ^2^	SCC SD ^2^
*Staphylococcus aureus* (*n* = 100)	Clinical IMI	18	10^5^–10^6^	>1 million	2.18 million	1,582,000
	Subclinical IMI	70	10^3^–10^4^	200,000–500,000	1.00 million	1,213,000
	Colonizer	12	10^3^–10^4^	≤100,000	45,000	33,000
Coagulase-negative staphylococci (*n* = 100)	Clinical IMI	18	10^3^–10^4^	>1 million	1.90 million	1,524,000
	Subclinical IMI	50	10^3^–10^4^	500,000–1 million	0.87 million	1,045,000
	Colonizer	32	10^3^–10^4^	≤100,000	65,000	50,000

*n* = number of isolates; cfu = colony forming units; SCC = somatic cell count; SD = standard deviation; IMI = intramammary infection. ^1^ Unit: cfu/mL; ^2^ Unit: cells/mL.

**Table 2:** Minimal inhibitory concentration of *Staphylococcus aureus* (*n* = 100) isolated from bovine mastitis in Austria.

	Antimicrobials										
MIC (µg/mL)	Penicillin G	Ampicillin	Cefazolin	Cefoperazone	Cefquinome	Oxacillin	Pirlimycin	Erythromycin	Amoxicillin/Clavulanic Acid 2:1	Kanamycin/Cephalexin 10:1	Marbofloxacin
0.125	90	NA	NA	NA	NA	NA	NA	0	NA	NA	NA
0.25	2	NA	NA	NA	NA	NA	NA	5	NA	NA	56
0.5	1	NA	NA	NA	NA	NA	NA	54	NA	NA	38
1	1	NA	NA	NA	93	95	31	25	NA	NA	4
2	1	NA	NA	90	4	3	28	5	NA	NA	1
4	3	95	96	6	3	0	14	7	100	87	NA
8	1	3	3	2	0	NA	NA	NA	0	13	NA
16	NA	2	1	2	NA	NA	NA	NA	0	0	NA
32	NA	NA	0	NA	NA	NA	NA	NA	0	0	NA
>(growth at highest concentration)	1	0	0	0	0	2	27	4	0	0	1
*S. aureus* ATCC 25923	≤0.125	≤4	≤4	≤2	≤1	≤1	≤1	0.5	≤4/2	≤4/0.4	≤0.25
*S. aureus* ATCC 29213	1	≤4	≤4	≤2	≤1	≤1	≤1	0.5	≤4/2	≤4/0.4	≤0.25
*S. aureus* ATCC 35556	≤0.125	≤4	≤4	≤2	≤1	≤1	2	0.25	≤4/2	≤4/0.4	≤0.25
*S. aureus* ATCC BAA-39	>8	>16	≤4	16	2	>4	>4	>4	≤4/2	>32/3.2	>2
MIC50	≤0.125	≤4	≤4	≤2	≤1	≤1	2	0.5	≤4/2	≤4/0.4	≤0.25
MIC90	≤0.125	≤4	≤4	≤2	≤1	≤1	>4	4	≤4/2	8/0.8	0.5
S (%)	90.00 *	NA	NA	90.00	NA	98.00	59.00	59.00	NA	100.00	NA
Breakpoints	S ≤ 0.12; R ≥ 0.25 ^a^	NA	NA	S ≤ 2; I = 4; R ≥ 8 ^b^	NA	S ≤ 2; R ≥ 4 ^a^	S ≤ 2; R ≥ 4 ^a^	S ≤ 0.5; I = 1–4; R ≥ 8 ^a^	NA	S ≤ 8; I = 16; R ≥ 32 ^c^	NA
Target pathogens	Human *Staphylococcus* spp.			Cattle *Staphylococcus* spp.		Human *S. aureus*	Cattle *S. aureus*	Human *Staphylococcus* spp.		Cattle *S. aureus*	

MIC = minimal inhibitory concentration; NA = not applicable. ^a^ CLSI VET08 2018 [21]; ^b^ Feßler et al., 2012 [20]; ^c^ Pillar et al., 2009 [19]. * Test according to CLSI: 6 of the 10 resistant isolates are producing β-lactamase. Green S (susceptible); Yellow I (intermediately susceptible); Red R (resistant).

**Table 3:** Distribution of coagulase-negative staphylococci species in relation to their presentation.

Species	Clinical IMI (*n*)	Subclinical IMI (*n*)	Colonizer (*n*)	Total (*n*)
*S. xylosus*	4	20	16	40
*S. chromogenes*	7	11	8	26
*S. haemolyticus*	1	3	3	7
*S. sciuri*	0	4	1	5
*S. saprophyticus*	0	1	2	3
*S. simulans*	1	1	1	3
*S. succinus*	1	2	0	3
*S. epidermidis*	0	1	0	1
*S. equorum*	0	1	0	1
*S. hyicus*	1	0	0	1
*S. intermedius*	1	0	0	1
*Staphylococcus* spp.	2	6	1	9

*S.* = *Staphylococcus*; IMI = intramammary infection; *n* = number of isolates.

**Table 4:** Minimal inhibitory concentration of coagulase-negative staphylococci (*n* = 100) isolated from bovine mastitis in Austria.

MIC (µg/mL)	Antimicrobials										
	Penicillin G	Ampicillin	Cefazolin	Cefoperazone	Cefquinome	Oxacillin	Pirlimycin	Erythromycin	Amoxicillin/ClavulanicAcid 2:1	Kanamycin/Cephalexin 10:1	Marbofloxacin
0.125	83	NA	NA	NA	NA	NA	NA	0	NA	NA	NA
0.25	5	NA	NA	NA	NA	NA	NA	12	NA	NA	35
0.5	4	NA	NA	NA	NA	NA	NA	46	NA	NA	47
1	3	NA	NA	NA	94	91	80	30	NA	NA	16
2	0	NA	NA	78	5	4	5	3	NA	NA	2
4	1	93	100	18	0	1	4	2	100	98	NA
8	0	4	0	3	0	NA	NA	NA	0	1	NA
16	NA	0	0	1	NA	NA	NA	NA	0	0	NA
32	NA	NA	0	NA	NA	NA	NA	NA	0	1	NA
>(growth at highest concentration)	4	3	0	0	1	4	11	7	0	0	0
*S. epidermidis* ATCC 12228	>8	8	≤4	≤2	≤1	≤1	≤1	0.5	≤4/2	≤4/0.4	≤0.25
*S. intermedius* ATCC 29663	≤0.125	≤4	≤4	≤2	≤1	≤1	≤1	0.5	≤4/2	≤4/0.4	≤0.25
*S. sciuri* ATCC 29060	0.25	≤4	≤4	4	≤1	2	2	0.5	≤4/2	≤4/0.4	1
*S. xylosus* ATCC 29971	≤0.125	≤4	≤4	4	≤1	2	≤1	0.5	≤4/2	≤4/0.4	1
*S. chromogenes* MIC90 (*n* = 26)	0.5	8	≤4	≤2	≤1	≤1	>4	4	≤4/2	≤4/0.4	0.5
S (%)	84.62	NA	NA	100.00	NA	NA	NA	38.46	NA	100.00	NA
*S. xylosus* MIC90 (*n* = 40)	0.5	≤4	≤4	4	≤1	2	4	1	≤4/2	≤4/0.4	1
S (%)	82.50	NA	NA	72.50	NA	NA	NA	65.00	NA	100.00	NA
Remaining CNS MIC90 (*n* = 34)	0.25	≤4	≤4	4	2	2	>4	2	≤4/2	≤4/0.4	1
S (%)	82.35	NA	NA	67.65	NA	NA	NA	64.70	NA	97.06	NA
Total MIC50 (*n* = 100)	≤0.125	≤4	≤4	≤2	≤1	≤1	≤1	0.5	≤4/2	≤4/0.4	0.5
Total MIC90 (*n* = 100)	0.5	≤4	≤4	4	≤1	≤1	>4	2	≤4/2	≤4/0.4	1
S (%)	83.00	NA	NA	78.00	NA	NA	NA	58.00	NA	98.00	NA
Breakpoints	S ≤ 0.12; R ≥ 0.25 ^a,^*	NA	NA	S ≤ 2; I = 4; R ≥ 8 ^b^	NA	S ≤ 0.25; R ≥ 0.5 ^a,^**	NA	S ≤ 0.5; I = 1–4; R ≥ 8 ^a^	NA	S ≤ 8; I = 16; R ≥ 32 ^c^	NA
Target pathogens	Human *Staphylococcus* spp.			Cattle *Staphylococcus* spp.		Human CNS except *S. lugdunesis*		Human *Staphylococcus* spp.		Cattle *Staphylococcus* spp.	

MIC = minimal inhibitory concentration; CNS = coagulase-negative staphylococci; NA = not applicable. ^a^ CLSI VET08 2018 [21]; ^b^ Feßler et al., 2012 [20]; ^c^ Pillar et al., 2009 [19]. * Test according to CLSI: 10 of the 100 isolates are producing β-lactamase. ** MIC criteria may overcall resistance for some CNS from bovine mastitis. Green S (susceptible); Yellow I (intermediately susceptible); Red R (resistant).

**Table 5:** Staphylococcal mastitis—Treatment approaches of veterinarians in Austria in response to culture results (multiple answers possible).

**Subclinical Mastitis and Control Examinations (Except Controls Prior to Drying-off)**	**CNS**	***S. aureus***
Intramammary administration of antimicrobials according to susceptibility testing	44.1%	22.1%
Also, parenteral administration of antimicrobials and a combination of parenteral and intramammary	28.7%	37.5%
SCC check and treatment in case of elevation	42.6%	26.5%
Generally, no antimicrobial treatment during lactation in case of subclinical mastitis	18.4%	39.7%
Other treatment except antimicrobials	9.6%	17.6%
Responding veterinarians (*n*)	136	136
**Control Prior to Drying-off**	**CNS**	***S. aureus***
Intramammary and parenteral administration of antimicrobials according susceptibility testing	19.1%	36.3%
Antimicrobial dry cow therapy	86.0%	81.5%
SCC check and treatment in case of elevation	17.6%	5.2%
Other treatment except antimicrobials	2.9%	13.3%
Responding veterinarians (*n*)	136	135

SCC = somatic cell count. CNS = coagulase-negative staphylococci. *S. aureus* = *Staphylococcus aureus*.

## Supplementary Materials

The following are available online at https://www.mdpi.com/2076-2615/9/5/200/s1, Table S1: Questionnaire.
