# Supplementary material for: Characterization of Staphylococcus Species Isolated from Bovine Quarter Milk Samples
Source: Animals (Basel). 2019 Apr 27;9(5):200. doi: 10.3390/ani9050200 (PMC6563082; doi:10.3390/ani9050200)
Supplement: Supplementary file 1 [file animals-09-00200-s001.pdf]

animals

MDPI

Table S1. Questionnaire.

CNS = coagulase-negative staphylococci; *S.* = *Staphylococcus*; SCC = somatic cell count

For immediate treatment of clinical mastitis I include

a) intramammary administration of antimicrobials
b) parenteral administration of antimicrobials
c) intramammary and parenteral administration of antimicrobials
d) other treatment except antimicrobials
e) substances used: ________________

In case of subclinical mastitis and control examinations (except controls prior to drying-off) with CNS positive culture results, my approach is

a) intramammary administration of antimicrobials according to susceptibility testing
b) also parenteral administration of antimicrobials and a combination of parenteral and intramammary
c) to check SCC and treat in case of elevation
d) generally no antimicrobial treatment during lactation in case of subclinical mastitis with CNS
e) other treatment except antimicrobials
f) substances used: ________________

In case of controls prior to drying-off with CNS positive culture result, my approach is

a) intramammary and parenteral administration of antimicrobials according to susceptibility testing
b) antimicrobial dry cow therapy
c) to check SCC and to treat in case of elevation
d) other treatment except antimicrobials
e) substances used: ________________

In case of subclinical mastitis and control examinations (except controls prior to drying-off) with *S. aureus* positive culture results, my approach is

a) intramammary administration of antimicrobials according to susceptibility testing
b) also parenteral administration of antimicrobials and a combination of parenteral and intramammary
c) to check SCC and treat in case of elevation
d) generally no antimicrobial treatment during lactation in case of subclinical mastitis with *S. aureus*
e) other treatment except antimicrobials
f) substances used: ________________

In case of controls prior to drying-off with *S. aureus* positive culture result, my approach is

a) intramammary and parenteral administration of antimicrobials according to susceptibility testing
b) antimicrobial dry cow therapy
c) to check SCC and to treat in case of elevation
d) other treatment except antimicrobials
e) substances used: ________________

Further comments
Free-text section
